# Patient and Health System Experience With Implementation of an Enterprise-Wide Telehealth Scheduled Video Visit Program: Mixed-Methods Study

**DOI:** 10.2196/medinform.8479

**Published:** 2018-02-13

**Authors:** Rhea E Powell, Danica Stone, Judd E Hollander

**Affiliations:** ^1^ Thomas Jefferson University Philadelphia, PA United States

**Keywords:** telemedicine, video visit, primary care, specialty, patient satisfaction

## Abstract

**Background:**

Real-time video visits are increasingly used to provide care in a number of settings because they increase access and convenience of care, yet there are few reports of health system experiences.

**Objective:**

The objective of this study is to report health system and patient experiences with implementation of a telehealth scheduled video visit program across a health system.

**Methods:**

This is a mixed methods study including (1) a retrospective descriptive report of implementation of a telehealth scheduled visit program at one large urban academic-affiliated health system and (2) a survey of patients who participated in scheduled telehealth visits. Health system and patient-reported survey measures were aligned with the National Quality Forum telehealth measure reporting domains of access, experience, and effectiveness of care.

**Results:**

This study describes implementation of a scheduled synchronous video visit program over an 18-month period. A total of 3018 scheduled video visits were completed across multiple clinical departments. Patient experiences were captured in surveys of 764 patients who participated in telehealth visits. Among survey respondents, 91.6% (728/795) reported satisfaction with the scheduled visits and 82.7% (628/759) reported perceived quality similar to an in-person visit. A total of 86.0% (652/758) responded that use of the scheduled video visit made it easier to get care. Nearly half (46.7%, 346/740) of patients estimated saving 1 to 3 hours and 40.8% (302/740) reported saving more than 3 hours of time. The net promoter score, a measure of patient satisfaction, was very high at 52.

**Conclusions:**

A large urban multihospital health system implemented an enterprise-wide scheduled telehealth video visit program across a range of clinical specialties with a positive patient experience. Patients found use of scheduled video visits made it easier to get care and the majority perceived time saved, suggesting that use of telehealth for scheduled visits can improve potential access to care across a range of clinical scenarios with favorable patient experiences.

## Introduction

Telehealth video visits, or real-time remote face-to-face visits between patients and providers, have been implemented in a number of settings in recent decades. Video visits have a well-established track record of use in rural and health shortage service areas, where the availability of providers may be limited [1,2]. Applications of various forms of telehealth including video visits have been studied in a number of settings, including behavioral health care [3], dermatology [4], genetic counseling [5], rheumatology [6], and pain management [7]. Real-time remote video visits have been shown to be an acceptable alternative to patients and providers in a number of settings and have the potential to reduce costs [8,9].

Although telehealth video visit use for scheduled routine visits are increasingly implemented in various health care settings, there are few published reports of health system experiences implementing telehealth programs that include scheduled video visits. In the United States, some information is available about system-wide implementation of clinical video telemedicine programs from the Veteran’s Administration (VA) [8]. The VA has reported experiences with cost savings related to widespread video visit use [10], which can inform other health systems seeking to implement system-wide video visit programs. A number of cost and reimbursement factors are unique to the VA setting, however, and may not be shared by other US health systems looking to adopt video visit programs. Other experiences can be gleaned from the international community. One health system report from a tertiary hospital in Australia described processes and outcomes of introducing of a centralized coordination for telehealth service [11], with a resulting increase in availability of telehealth services. This work focuses primarily on health system factors, and does not include patient experiences of implementation. An understanding of health system and patient experiences with implementation of telehealth visits is needed to improve design and delivery of telehealth for scheduled visits.

We report experiences of one large urban health system in implementing an enterprise-wide scheduled video visit program across various disciplines and specialties, with a focus on the impact on access, experience, and effectiveness of care. These three domains of care represent three of the four domains that inform the National Quality Forum (NQF) telehealth measures framework [12].

## Methods

### Study Design and Setting

This is a mixed methods study evaluating the JeffConnect scheduled visit program at a large urban academic-affiliated health system, Jefferson Health (Jefferson), located in Philadelphia, PA, USA. The study includes a retrospective descriptive evaluation of implementation of the program and a survey of patients who participated in scheduled telehealth visits.

### Selection of Survey Participants

Patients aged 18 years and older with an existing relationship with a Jefferson provider who was trained on telehealth use were eligible to participate in scheduled telehealth visits. Patients were informed of the option of a telehealth scheduled visit by their provider, an administrator, or learned about it through marketing notifications. All patients who participated in a telehealth visit were eligible to participate in the survey. Patients were contacted by email the second week following their scheduled visit to provide feedback via survey.

### Program Description

Jefferson Health provides hospital-based and outpatient-based services to patients across its four hospital systems, including the academic medical center at Thomas Jefferson University. In 2015, Jefferson initiated JeffConnect, an enterprise-wide telehealth program that offers video visits with a Jefferson health care provider via Web or mobile app, allowing patients to follow up with providers virtually as an alternative to returning to the office in-person. Patients and providers schedule appointments the same way they would for in-person visits, and visits are performed via real-time face-to-face remote video.

The JeffConnect team comprises a program and project manager and five telehealth coordinators. The telehealth coordinators are trained to be responsible for coordinating clinical services and enhancing patient engagement via telehealth. Telehealth coordinators are not medical providers. They are college educated and have completed an American Telehealth Association-accredited telehealth facilitator certificate program [13]. Coordinators use videoconferencing technologies and scheduling software to coordinate and connect staff, patients, and providers in the manner effective to delivery of services, patient care, education, and training. Scheduled video visits were initially piloted with the institution’s covered employees who are also patients, then offered widely to all established Jefferson patients in all specialties.

### Training Program

During its initial implementation phase, the JeffConnect team conducted more than 50 two-hour in-person group education sessions training providers, schedulers, and staff. The training session covered topics including program description, legal and regulatory information relevant to providing care via telehealth, how to use the telehealth platform for conducting video visits, and value to patients. Presently, the in-person group telehealth classes are now individually offered virtually through webinar.

### Scheduling Visits

Patients schedule video visits using the same processes that are used for scheduling in-person visits, either by calling a centralized health system scheduler, calling the office, or by requesting a telehealth visit online. Many patients were referred to schedule via telehealth by their provider, and appropriateness for telehealth visit was determined by provider for all visits. All patients at the health system receive an automated reminder phone call 2 days prior to the visit, which is in place for on-site and telehealth visits. Additionally, for telehealth visits, the patient receives a phone call the day before from a telehealth coordinator to review processes for log-on, check that any necessary steps such as app download and registration are completed, and test the connection.

### Conducting Visits

On the day and time of the scheduled video visit, patients log on to their password-protected JeffConnect account using a mobile phone or tablet app, or via a laptop or desktop browser equipped with a webcam and microphone. Providers log on from their health system location using a tablet app or Web browser, also with a webcam and microphone. Visits include real-time video and audio. Providers have access to the electronic medical record to review the patient’s prior records and document the visit.

### Data Collected

Data were collected with a focus on the impact on access, experience, and effectiveness of care, because these three domains of care represent three of the four domains that inform the NQF telehealth measures framework [12].

### Access

Health system measures of access to care via telehealth scheduled visit included number of providers trained to use telehealth for scheduled visits, the number of downloads and registrations of the app, and the number of completed visits. Metrics for each department were collected and reported monthly, indicating how many visits were completed and by which provider. Providers were categorized by specialty, including dermatology, emergency medicine, family medicine, medical subspecialties (allergy, cardiology, endocrinology, gastroenterology, hematology, infectious disease, nephrology, oncology, pulmonology, and rheumatology), neurology, obstetrics and gynecology, psychiatry, radiation oncology, radiology, rehabilitation medicine, and surgical and related subspecialties (anesthesia, general surgery, neurosurgery, oral maxillofacial surgery, otolaryngology, preadmission testing, and urology).

Patient-reported measures of access to care included reported ease of use and impact on the ability to receive care when and where needed (both on a five-point Likert scale), as well as patient estimates of time saved through use of the telehealth visit.

### Experience

Patient experience was assessed using a series of questions including overall patient satisfaction, reasons for dissatisfaction (if noted), if the patient would use JeffConnect for a scheduled telehealth visit again, and if the patient would recommend it to a family member or friend. Experience was also assessed through calculation of net promoter score, a measure of willingness to recommend to others.

### Effectiveness

Effectiveness of care was assessed using health system data including qualitative responses from division directors. Patient responses relevant to effectiveness of care included patients’ perspectives of whether level of care received via telehealth was equal to level of care received via in-person visit, and whether patients had adequate time with the provider, assessed on five-point Likert scale.

### Data Analysis

Data are presented descriptively as absolute numbers and percent frequency of occurrence.

## Results

### Access

The Jefferson telehealth program trained 746 providers, including physicians and advanced practice providers, to perform scheduled video visits. A summary of total completed visits between January 2015 and December 2016 are presented in Table 1.

All the clinical care departments that provide outpatient care had physicians capable of delivering telehealth. There were 32,234 registrations and downloads of the JeffConnect app, and 3018 scheduled outpatient video visits were completed during the 18-month implementation period.

Of the 3018 completed video visits, 764 patients responded to the after-visit survey. Patient survey responses are summarized in Table 2. Most patients (84.8%, 646/762) surveyed had no prior experience with telehealth video visits. The majority (86.0%, 652/758) agreed or strongly agreed that JeffConnect made it easier to get care.

### Experience

Among survey participants, 91.3% (728/797) reported satisfaction with their scheduled telehealth video visit. Among the 67 participants who reported they were not satisfied, 57 of those cited technical issues and five reported they did not like interacting on video. A total of 86.7% (656/757) agreed or strongly agreed it was easy to use and 90.9% (686/755) would use it again. The net promoter score, a reflection of patient willingness to recommend scheduled visits, was 52, consistent with high likelihood of recommending the service.

**Table 1:** Scheduled video visits completed from January 2015 to December 2016 by department.

Department	Visits by physicians, n	Visits by advanced practice providers, n
Dermatology	32	3
Emergency medicine	88	0
Family medicine	32	0
Medical subspecialties	734	233
Neurology	10	0
Obstetrics & gynecology	40	9
Psychiatry	240	40
Radiation oncology	55	5
Radiology	60	0
Rehabilitation medicine	50	0
Surgical subspecialties	908	479
Total	2249	769

**Table 2:** Scheduled visit patient survey responses (N=764).

Question and response		n (%)	
**How did you hear about JeffConnect?**			
	Email		42 (5.2)
	Postal mail		1 (0.1)
	Friend or family		24 (3.0)
	Health care provider		554 (69.7)
	Jefferson website		39 (4.9)
	Print advertisement		5 (0.6)
	Online advertisement		1 (0.1)
	Other		129 (16.2)
**Have you ever had a telehealth video visit before this visit?**			
	Yes		116 (15.2)
	No		646 (84.8)
**Do you use social media?**			
	Yes		63 (71.6)
	No		25 (28.4)
**Have you recommended JeffConnect to your friends or family?**			
	Yes		307 (43.6)
	No		397 (56.4)
**Overall, were you satisfied with your most recent visit?**			
	Yes		728 (91.6)
	No		67 (8.4)
**What is the reason you were unsatisfied with your visit (check all that apply)^a^**			
	I experienced technical issues		53 (83.1)
	I didn’t like interacting on video		5 (7.9)
	I was not happy with the physician		0 (0.0)
	Other		27 (42.8)
**How much time do you think JeffConnect saved you?**			
	None		31 (4.1)
	Less than 1 hour		61 (10.7)
	1-3 hours		346 (45.5)
	More than 3 hours		302 (39.7)

^a^Respondents had the option to identify more than one response.

### Effectiveness

Use cases for scheduled visits varied by department. Many of the clinical departments used scheduled video visits for routine follow-up to assess an ongoing episode of care, chronic condition management, medication updates, and to engage families in outpatient care. Anesthesiology used scheduled video visits for some components of preadmission testing before surgery and for postoperative pain management. Surgical specialties (urology, otolaryngology, and oral maxillofacial surgery) employed scheduled telehealth visits for postop follow-up. Rehabilitation medicine used telehealth scheduled video visits for transitions of care visits after hospital discharge, wound care visits, prosthesis monitoring, and physical therapy follow-up. Obstetrics and gynecology use cases included family planning visits.

Among patient responses with regard to effectiveness, 91.0% (691/759) reported having had enough time with the provider and 82.7% (628/759) perceived the same level of care as in in-person visits. More than 87.6% (648/740) perceived at least 1 hour of time saved by converting outpatient visit to a scheduled telehealth visit, and nearly 40.8% (302/740) perceived more than 3 hours of time saved.

## Discussion

This study reports the initial implementation of a scheduled video visit program at one large academic health system, including completion of 3018 scheduled telehealth visits across all clinical departments in the enterprise. Our findings demonstrate that use of telehealth for scheduled visits increases potential and realized access to health care across a range of clinical scenarios, and is associated with favorable patient experiences.

The NQF report establishing a framework for measuring quality of care provided through telehealth focuses on access, experience, effectiveness, and financial impact of care [12]. Access to care includes access for patients and families, access for the care team, and access to information. The Anderson and Aday [14] conceptual model for understanding access to care considers potential (resources that allow patients to seek care) and realized access (actual use of care). This study provides input on the ability of a large health system to increase potential access to care by enabling providers in every clinical department to potentially provide care and facilitating availability of telehealth scheduled visits to patients who have registered and downloaded the app. The study also demonstrates the impact on realized access to care across a range of clinical departments through the completed visits.

Improving access to health care has been touted as a primary value added by telehealth in health care [15-17], and policy recommendations for improving access to care include integrating telehealth into care [18]. We add to the existing body of literature around improved access through telehealth with evidence of a large health system’s experience implementing scheduled video visits into routine care of existing patients. Measuring access to care under the NQF framework for telehealth will importantly include access for patients and family, and will also include access for the care team and access to information (electronic health records and health information). Although this study does not directly evaluate access for the care team or access to information, we note that clinicians providing care via scheduled video visit have continuous access to the electronic medical record while engaging in the video visit.

This work also builds on existing literature suggesting that patient’s report favorable experience with telehealth video visits [7,19,20]. We add to this work, and add to it with the use of the net promoter score, to assess patient satisfaction with telehealth services. The net promoter score is a metric to estimate how likely an individual is to recommend a service. Initially used in marketing [21,22], and more recently adapted for use in health care [23], the net promoter score allows for categorization of survey respondents either as a “promoter,” “passive,” or “detractor.” The score is calculated by the percentage of promoters minus the percentage of detractors, and ranges from –100 to 100. A positive score of 52, such as we found among our patients, reflects high likelihood of recommending to friends and family.

There is a broad and growing body of literature surrounding the impact of telehealth video visits on access and experience of care, but the effectiveness of telehealth scheduled video visits for routine care and the financing of scheduled video visits are incompletely understood. The clinical use cases reported were wide-ranging and varied significantly by department, making a uniform assessment of quality of care provided challenging. These findings demonstrate that the majority of patients surveyed across a heterogeneous group of clinical scenarios felt they had received the same level of quality as they would have during on-site in-person visits.

### Limitations

This study reports on experiences with initial implementation of an enterprise-wide scheduled video visit program. Patients who participated in scheduled visits self-selected to use video visits to connect with their providers. Although very few of these patients had any prior experience with video visits for health care, they were nevertheless the early adopters of this application of telehealth at our health system. Their perceptions may not be generalizable to other populations who did not choose to use telehealth.

Additionally, the perspective of providers and staff are not captured by these data. Provider and staff engagement are essential to the success of a system-wide program. Implementation of comprehensive scheduled video visit programs require communicating the value of telehealth to providers, compensating accordingly, keeping information technology applications and workflows simple, recognizing the workload that providers handle, and investing in a culture where providers are trained and rewarded for providing high-quality care that includes telehealth visits [24]. Future work should address the experience of the care team in widespread implementation of scheduled video visits.

Finally, we were unable to evaluate the financing of a scheduled video visit program with this work. During our initial implementation period, telehealth scheduled visits were not compensated by most payers and patients were not billed for this uncovered benefit; as such, evaluating the financial implications was not possible. Health systems considering system-wide implementation of telehealth program should identify motivations and barriers of all stakeholders for telehealth scheduled visits among patients, providers, administrators, and payers [25], and they will need information on how a scheduled visit program impacts care access, experience, effectiveness, and financing.

### Conclusions

Health care delivery is in a state of flux, shifting from traditional in-person, visit-based, fee-for-service models toward care delivery that is patient-centered, efficient, and lower cost. Effective use of telehealth video visits can facilitate meeting these goals, but requires broad adoption and integration into clinical care. Our experiences implementing an enterprise-wide telehealth program at one large urban multihospital health system demonstrate the promise that scheduled telehealth video visits hold for improving access, supporting a positive patient experience and providing effective care.

## Abbreviations

NQF: National Quality Forum
VA: Veteran’s Administration
